# Introducing a Comprehensive Framework to Measure Spike-LFP Coupling

**DOI:** 10.3389/fncom.2018.00078

**Published:** 2018-10-15

**Authors:** Mohammad Zarei, Mehran Jahed, Mohammad Reza Daliri

**Affiliations:** ^1^Department of Electrical Engineering, Sharif University of Technology, Tehran, Iran; ^2^School of Electrical Engineering, Iran University of Science and Technology, Tehran, Iran; ^3^School of Cognitive Sciences, Institute for Research in Fundamental Sciences (IPM), Tehran, Iran

**Keywords:** local field potentials, phase locking value, spike field coherence, pairwise phase consistency, spike-LFP phase coupling

## Abstract

Measuring the coupling of single neuron's spiking activities to the local field potentials (LFPs) is a method to investigate neuronal synchronization. The most important synchronization measures are phase locking value (PLV), spike field coherence (SFC), pairwise phase consistency (PPC), and spike-triggered correlation matrix synchronization (SCMS). Synchronization is generally quantified using the PLV and SFC. PLV and SFC methods are either biased on the spike rates or the number of trials. To resolve these problems the PPC measure has been introduced. However, there are some shortcomings associated with the PPC measure which is unbiased only for very high spike rates. However evaluating spike-LFP phase coupling (SPC) for short trials or low number of spikes is a challenge in many studies. Lastly, SCMS measures the correlation in terms of phase in regions around the spikes inclusive of the non-spiking events which is the major difference between SCMS and SPC. This study proposes a new framework for predicting a more reliable SPC by modeling and introducing appropriate machine learning algorithms namely least squares, Lasso, and neural networks algorithms where through an initial trend of the spike rates, the ideal SPC is predicted for neurons with low spike rates. Furthermore, comparing the performance of these three algorithms shows that the least squares approach provided the best performance with a correlation of 0.99214 and *R*^2^ of 0.9563 in the training phase, and correlation of 0.95969 and *R*^2^ of 0.8842 in the test phase. Hence, the results show that the proposed framework significantly enhances the accuracy and provides a bias-free basis for small number of spikes for SPC as compared to the conventional methods such as PLV method. As such, it has the general ability to correct for the bias on the number of spike rates.

## Introduction

Synchronous neural activity plays an important role in brain studies (Galarreta and Hestrin, 2001; Tallon-Baudry et al., 2004; Uhlhaas et al., 2009; Pipa and Munk, 2011; van Wijk et al., 2012; Mendoza-Halliday et al., 2014; Seif and Daliri, 2015; Wong et al., 2016). Neural activities are based on either spikes or local field potentials (LFPs). LFPs reflect the activity of a population of neurons based on spatially averaged synaptic activity (Buzsáki and Draguhn, 2004; Buzsáki et al., 2012). An appropriate approach to study rhythmic neuronal synchronization is provided by relating spiking activities to the oscillations of the ongoing LFPs (Salinas and Sejnowski, 2001; Pikovsky et al., 2002; Tiesinga et al., 2008; Fries, 2009). The relation between the spiking activities and the oscillations of LFPs is a helpful way to evaluate the rhythmic neuronal synchronization for a given frequency band. As such, spike-LFP phase coupling (SPC) should account for cell type and firing rate specification, must allow for structured rhythmic activity and should avoid volume conduction complications (Canolty et al., 2010; Hoerzer et al., 2010; Vinck et al., 2010, 2012; Denker et al., 2011; Hansen and Dragoi, 2011; Xu et al., 2013; Herreras, 2016).

Several spike-LFP synchronization measures have been introduced in previous studies, e.g., cross correlation and coherence coefficient, (Carter et al., 1973; Carter, 1987; Zeitler et al., 2006; Lepage et al., 2011; Srinath and Ray, 2014) phase synchronization or phase locking value (PLV) (Lachaux et al., 1999), spike field coherence (SFC) (Fries et al., 2001, 2002; Curtis et al., 2009; Grasse and Moxon, 2010; Hagan et al., 2012), pairwise phase consistency (PPC) (Vinck et al., 2010), and spike-triggered correlation matrix synchronization (SCMS) (Li et al., 2016).

Cross correlation and coherence coefficient methods are biased on the power and are not suitable for non-linear and non-stationary dynamics. In addition, coherence coefficient method overlooks the time resolution. Hence, the main synchronization measures are based on the PLV, SFC, PPC, and SCMS approaches. However, as we will discuss later, the PLV and the SFC methods are biased either on the spike rates or on the number of trials, which varies across trials or neurons.

The PLV measure suffers from an extreme dependency on the spike rates. Suppose that we have recorded spikes and the LFPs simultaneously from a neuron for 1 min. We first consider a 10 s interval and calculate the PLV for this time slot where an increase of the time interval results in a smaller average-vector for the PLV. It should be pointed out that as long as the number of included spikes within the time intervals are unchanged, the neuron's state remains unaffected. This observation illustrates that the PLV is biased on the spike rates. As a result, studies that use this method, equalize the spikes at a specified rate. Thus, to calculate the SPC by the PLV method, an equalizing scheme is used for the spikes based on a threshold T. The trials whose number of spikes are below T are deleted, and those with higher spikes than T, are equalized to T. As such, this method may eliminate large portion of the data.

Measuring SPC as a function of frequency is provided by the SFC method. SFC ranges between 0 and 1 for a given frequency. Therefore, if the SFC for a given frequency is 1, then all spikes associated with this frequency must have occurred with the same phase. On the other hand, if the SFC for a given frequency is 0, then the spike occurrence phase for spikes is considered dispersed and disassociated to the LFP component. Furthermore, the SFC is computed by comparing the magnitude of the frequency in the spike-triggered average (STA) and the average magnitude of the frequency in each of the LFP segments that are involved in the STA. As the STA is provided by the sum of the magnitude for all LFP segments, it is normalized by the spike rates. Additionally, the STA is quantified by the power spectral density, which specifies the strength of STA frequency components. Therefore, as the PSD of the STA is dependent on the PSD of the LFP, a spike which occurs during a high magnitude cycle of the LFP will have more impact on the value of the SFC than a spike which occurs during a low magnitude LFP cycle (Grasse and Moxon, 2010).

PPC computes the mean inter-spike resemblance of the LFP phase across all possible pairs of spikes. It computes the cosine of the absolute angular distance, namely the vector dot product for all given pairs of relative phases. It is claimed that the PPC is not affected by the bias on the spike rates (Vinck et al., 2010). However, the PPC based methods suffer from bursting and noise. They have high variance for low number of spikes and may result in negative values which is physiologically meaningless.

The key idea of the SCMS method is to consider the LFP segments placed in the center of each spike (spike-triggered LFPs) as multi-channel signals and to compute the index of the spike-LFP synchronization through constructing a correlation matrix. The method constructs the correlation matrix C through computing the PLV between pairs of the LFP segments. Furthermore, the eigenvalues of the correlation matrix is calculated and the maximum eigenvalue is reported as the coupling synchronization (Li et al., 2016). As a result, the SCMS measures the correlation in terms of phase in regions around the spikes not just at the moment of the spike occurrence which is its essential difference from the SPC. Also, Since the LFP signal is filtered in a specific band, it is not considered a single tone signal and there are probably different frequency components in each LFP segment.

Suppose that we have several spikes in a trial which occur close to 90° phase of LFP. Although, all the spikes of each LFP segment are in the same phase but because of their difference in frequency components, the vector of the phase difference of each pair differs. As such, the spikes will not be in the same phase although it was expected that the SCMS would reveal maximum synchronization. The aforementioned coupling bias points to a challenge which is frequently faced by neuroscientists. Therefore, to overcome the shortcomings of such coupling techniques one has to equalize the spike rates of different trials or neurons, which may discard important information.

In this study, a new framework is proposed to predict the SPC in trials with low number of spikes. This is done via modeling based on proper mathematical functions and by implementing machine learning algorithms. This new framework is presented in three phases. In the first phase, the ideal SPC is estimated using a model which is based on exponential functions. Briefly, the ideal SPC considers an infinite number of spikes by modeling the full spike estimation based on the PLV method. The second phase utilizes machine learning algorithms, namely least squares (Abdi, 2007), Lasso (Tibshirani, 1996, 1997) and neural networks (ELM) algorithms (Huang et al., 2006, 2012) where through an initial trend of the spike rates, the ideal SPC is predicted. The third phase presumes no systematic relationship between the spike rates and the corrected SPC. As it is neither strongly ascending nor descending, the estimated SPC is defined based on the first 20 points or spike rates of the trained model. Hence, it can be deduced that there may be no bias on the spike rates and the proposed framework may provide an alternative to the conventional methods such as the PLV. In what follows, the proposed method is introduced and the results based on experimental data are discussed.

## Materials and methods

Single-unit activity and the LFP signals were recorded from the MT area of the brains of macaque monkeys using a five-channel recording system discussed in a previous study (Seif and Daliri, 2015). Overall 100 sites were selected to be analyzed, where each site contained about 200 trials. The time duration of the analysis was 800 ms which started from 200 to 1,000 ms, measured based on the onset of the stimulus. The 50 Hz noise, and the LFPs in the 5–8 Hz band were band-pass filtered and removed using EEGLAB (Delorme and Makeig, 2004). The most important phase relation measure is the PLV which ranges between 0 and 1, as noted before. PLV method provides the magnitude of mean phase difference between the LFP and spikes as the strength of SPC.

The instantaneous LFP-phase is computed using the Hilbert transform. For each trial spike which corresponds to the LFP signal, the instantaneous LFP-phase to spike occurrence is calculated using the Hilbert transform. The Hilbert transform of a function *x*(*t*) denoted by *HT*(*x*(*t*)), converts a real-valued signal into a complex analytical signal from which the instantaneous phase can be extracted (Gabor, 1946; Boashash, 1992; Huang and Wu, 2008). The Hilbert transform is defined as,

$$\begin{array}{l} y\left(t\right)=HT\left(x\left(t\right)\right)=\frac{1}{\pi }P\overset{\infty }{\underset{-\infty }{\int }}\frac{x\left(\tau \right)}{t-\tau }\;d\tau \end{array}$$

where *P* is the Cauchy principal value of the singular integral. Using the above equation, the instantaneous phase, ϕ can be deduced from the analytical signal,

$$\begin{array}{l} \phi \left(t\right)=\text{arctan}\left(\frac{y\left(\text{t}\right)}{x\left(\text{t}\right)}\right) \end{array}$$

Also, the PLV method is defined by the following formula,

$$\begin{array}{l} PLV=\;\frac{1}{N}|\overset{N}{\underset{n=1}{\sum }}exp\left(j\varphi \right)|, \end{array}$$

where φ is the LFP phase at which the spike occurs, and *N* is the number of observations (Lachaux et al., 1999).

As noted before, the results of this study are obtained using three separate algorithms, namely least squares, Lasso, and ELM. These algorithms are briefly described below.

1. *Least squares regression*: This algorithm is a common technique used in data fitting modeling. Least squares regression is used as a primary approximation tool due to its efficiency and fullness where the square changes of the data are summed and minimized to find the best-fitting. Hence, for each sample data points (*x*_*i*_, *y*_*i*_), *i* = 1, …, *n* and the model function *f*(*x*, β), *r*_*i*_ is given by,

$$\begin{array}{l} r_{i}=y_{i}-f\left(x_{i},\beta \right). \end{array}$$

In order to obtain the optimal parameters, the least squares algorithms minimizes the following equation (Abdi, 2007),

$$\begin{array}{l} S=\overset{n}{\underset{i=1}{\sum }}r_{i}^{2}. \end{array}$$

2. *Machine learning least absolute shrinkage and selection operator (Lasso)*: Lasso is a regression analysis approach that utilizes regularization to improve the estimation accuracy. Lasso was originally stated in the framework of least squares. The lasso solves the *l*_1_-penalized regression problem of finding β to minimize,

$$\begin{array}{l} \underset{\beta_{0},\beta }{\min}\left(\frac{1}{2N}\overset{N}{\underset{i=1}{\sum }}{\left(y_{i}-\beta_{0}-x_{i}^{T}\beta \right)}^{2}+\lambda \overset{L}{\underset{j=1}{\sum }}|\beta_{j}|\right), \end{array}$$

where *i* = 1, 2, …, *N* and *j* = 1, 2, …, *L* that *N* is the total number of observations. The tuning parameter λ regulates the strength of the penalty. Each observation contains *L* covariates an d*y*_*i*_ is the *i*th outcome, *x*_*i*_ is the *i*th covariate vector, β is the L-vector, and β_0_ is a scalar. Letting *X* be the covariate matrix, so that *X*_*ij*_ = (_*x*_*i*_)*j*_ and $x_{i}^{T}$ is the *ith* row of *X*, the expression can be written more compactly as

$$\begin{array}{l} \underset{\beta_{0},\beta }{\min}\left\{\frac{1}{2N}||y-\beta_{0}1_{N}-X\beta |{|}_{2}^{2}\right\}\;\text{subject to }{\left||\beta |\right|}_{1}<t. \end{array}$$

This is equivalent to minimizing the sum of squares with a constraint of the form ||β||_1_<*t*. Here *t* is a free parameter that determines the amount of regularization. Because of the form of the *l*_1_-penalty, the lasso provides variable selection and shrinkage,

$$\begin{array}{l} {\hat{\beta }}_{0}=ȳ-{\bar{x}}^{T}\beta . \end{array}$$

This can be rewritten as,

$$\begin{array}{l} {\hat{\beta }}^{Lasso}=\underset{\beta }{\min}||y-X\beta |{|}_{2}^{2}+\lambda \overset{L}{\underset{j=1}{\sum }}|\beta_{j}|, \end{array}$$

where “${\hat{\beta }}^{Lasso}=$ the linear regression estimate” is obtained for λ = 0, and ${\hat{\beta }}^{Lasso}=$0 is attained for λ = ∞. Hence the values are within these two extremes, thereby fitting a linear model of *y* on *X* and shrinking the coefficients (Tibshirani, 1996, 1997).

3. *Extreme learning machine (ELM)*: ELM is feed forward neural network for regression. It can be used with a single or multi layers hidden nodes. In ELM, the hidden layer need not be tuned and the output function of ELM with *M* hidden nodes is given by,

$$\begin{array}{l} f_{M}\left(x\right)=\overset{M}{\underset{i=1}{\sum }}K_{i}h_{i}\left(x\right)=\;h\left(x\right)K, \end{array}$$

where *K*_*i*_ and *h*_*i*_(*x*) are the output weight and output function of the *ith* hidden node, respectively, *h*(*x*) is a feature mapping where *h*_*i*_(*x*) = *G*(*a*_*i*_, *b*_*i*_, *x*) with *G*(*a*_*i*_, *b*_*i*_, *x*) defined as the sigmoid function given by,

$$\begin{array}{l} G\left(a_{i},b_{i},x\right)=\frac{1}{1+\exp\left(-\left(a_{i}.x+b_{i}\right)\right)}, \end{array}$$

where *a*_*i*_ and *b*_*i*_ are hidden node parameters. ELM minimizes the training error and the output weights,

$$\begin{array}{l} \text{Minimize}:{\left||HK-T|\right|}^{2}\text{ and }||K||, \end{array}$$

where *H*and *T* are the following hidden-layer output matrix and *T* is the training data target matrix, respectively (Huang et al., 2006, 2012),

$$\begin{array}{l} H=\left[\begin{array}{c} h\left(x_{1}\right)\\ \vdots \\ h\left(x_{N}\right) \end{array}\right]=\left[\begin{array}{ccc} h_{1}\left(x_{1}\right) & \ldots & h_{L}\left(x_{1}\right)\\ \vdots & \vdots & \vdots \\ h_{1}\left(x_{N}\right) & \ldots & h_{L}\left(x_{N}\right) \end{array}\right],T=\left[\begin{array}{c} t_{1}\\ \vdots \\ t_{N} \end{array}\right]. \end{array}$$

## Results

### Estimation of the ideal SPC

**Figure 1:**
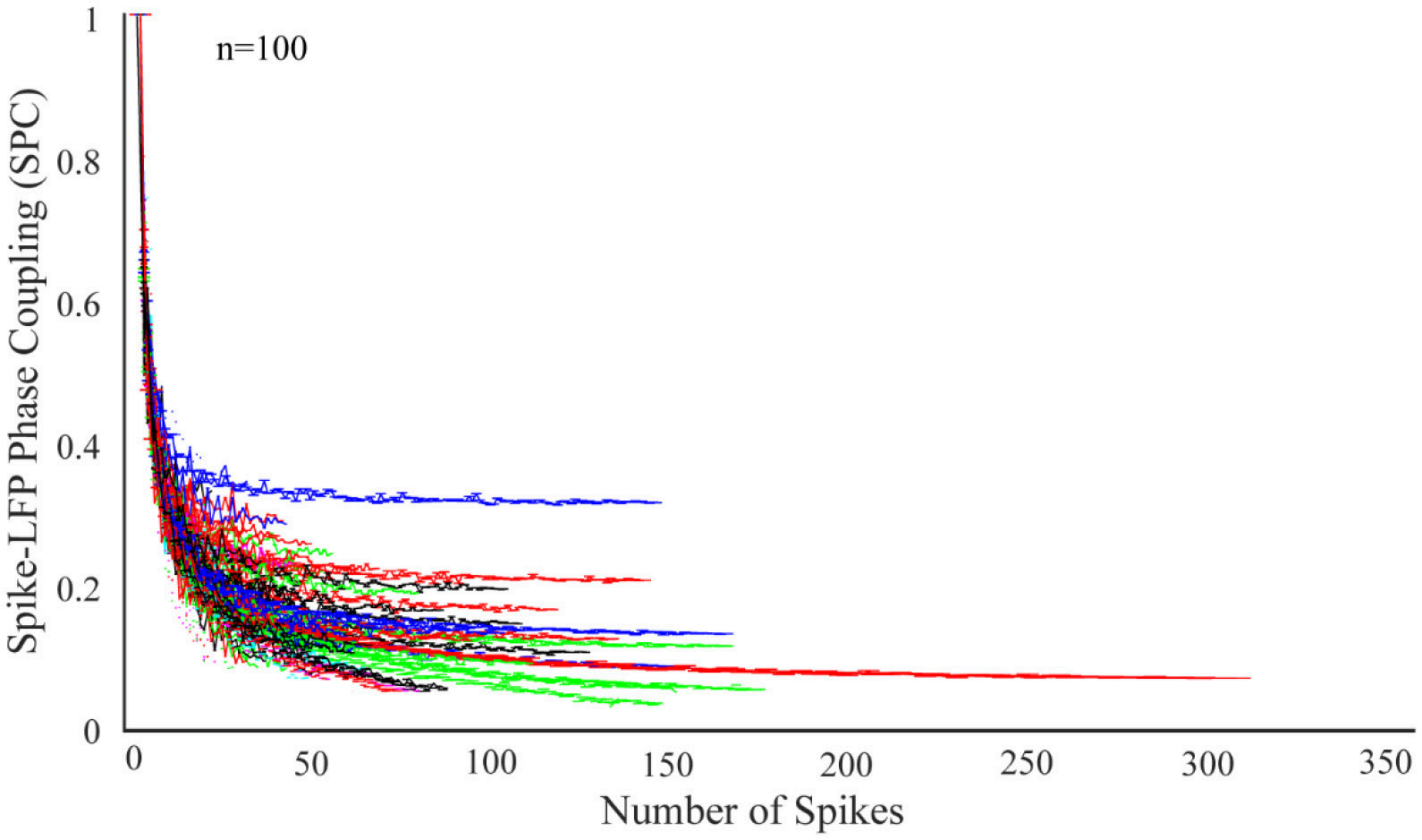
Dependence of the spike-LFP phase coupling (SPC) on the number of spikes for 100 neurons. SPC curves exhibit an exponential descending behavior and are biased on the spike rates.

In order to study the dependence of SPC on number of spikes, the first step is to check the exponential descending behavior of neurons. We first take 100 neurons and select those trials with the highest spikes or with the most information. Next, the SPC is measured using the PLV method for each of these trials. The ideal SPC considers an infinite number of spikes by modeling the full spike estimation based on the PLV method. The results are shown in Figure 1. The expression, “infinite number of spikes” actually refers to the asymptote of the fitted model for each of the SPC curves. In other words, the SPC value at the maximum rate of its spike is chosen as the SPC value. In fact, this SPC is based on the maximum information and is most closely related to the ideal SPC value. Based on the PLV method, the higher the number of spikes, the more accurate SPC will be. That is, as the number of spikes continue to decrease, PLV is less likely to yield the ideal SPC value. In fact, the ideal SPC occurs at its steady state value and its maximum spike rate for each trial, which is the same as the asymptote of the fitted model for each of the SPC curves.

Figure 1 illustrates SPC curves relative to the number of spikes for all neurons, depicting an exponential descending behavior which is biased on the spike rates. Therefore, the SPC value exponentially descends as the spike rate increases. All other neurons follow a similar pattern, confirming that acquiring SPC by the PLV method is highly dependent on the number of spikes. Therefore, generally and under any experimental setting, if SPC analyses are based on the PLV method, such exponentially descending behavior is expected.

**Figure 2:**
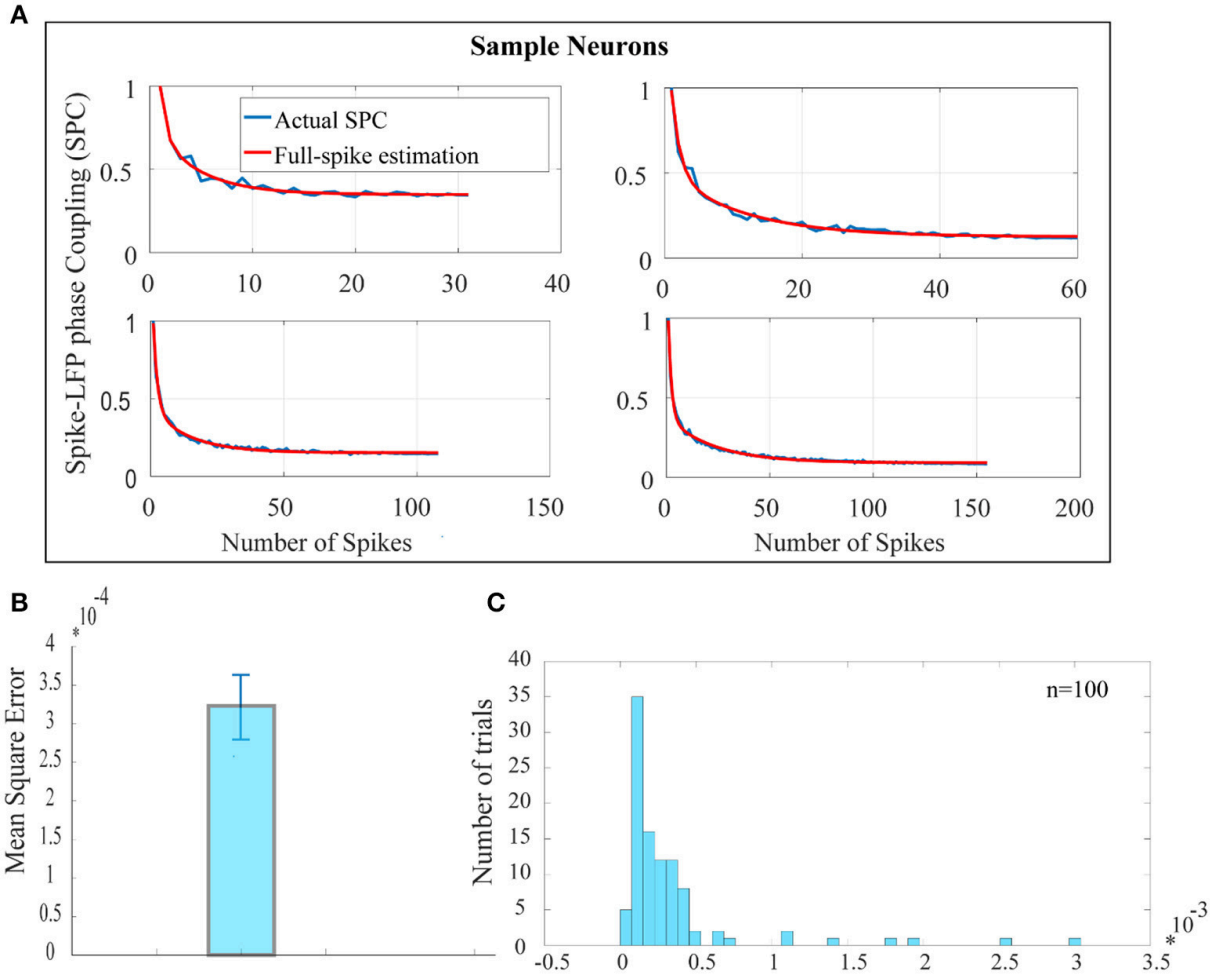
Estimation of the ideal SPC value using exponential modeling. **(A)** For a sample of 4 neurons, PLV based SPC exhibits an exponential descending behavior and is biased on the spike rates (the blue curves). The red curves show an estimated SPC through modeling with two exponential functions and an asymptote. **(B)** Depicts the average and error bar of RMSE across 100 trials among 100 neurons. The values are very small which illustrates high accuracy for estimation of SPC (0.0003±0.01). **(C)** Shows the distribution function of RMSE across 100 trials.

Figure 2A shows the SPC curves for a sample of 4 neurons. The blue curves in Figure 2A shows the SPC curves relative to the number of spikes measured by the PLV method and the red curves illustrates the model for fitting the SPC curves. The fitted model consists of two exponential functions and one constant term and is based on the total number of spikes or full spike estimation. The asymptote of the fitted model to each of the SPC curves shows the ideal SPC and illustrates their convergence. The root-mean-square error (RMSE) criteria shows that the fitted function is the best description for the SPC. RMSE is commonly used in regression analyses to verify experimental results. The average and error bar of RMSE for 100 trials among 100 neurons, populated across different neurons with highest spikes, is shown in Figures 2B,C depicts the distribution function of RMSE across 100 trials.

### Predicting a more reliable SPC

**Figure 3:**
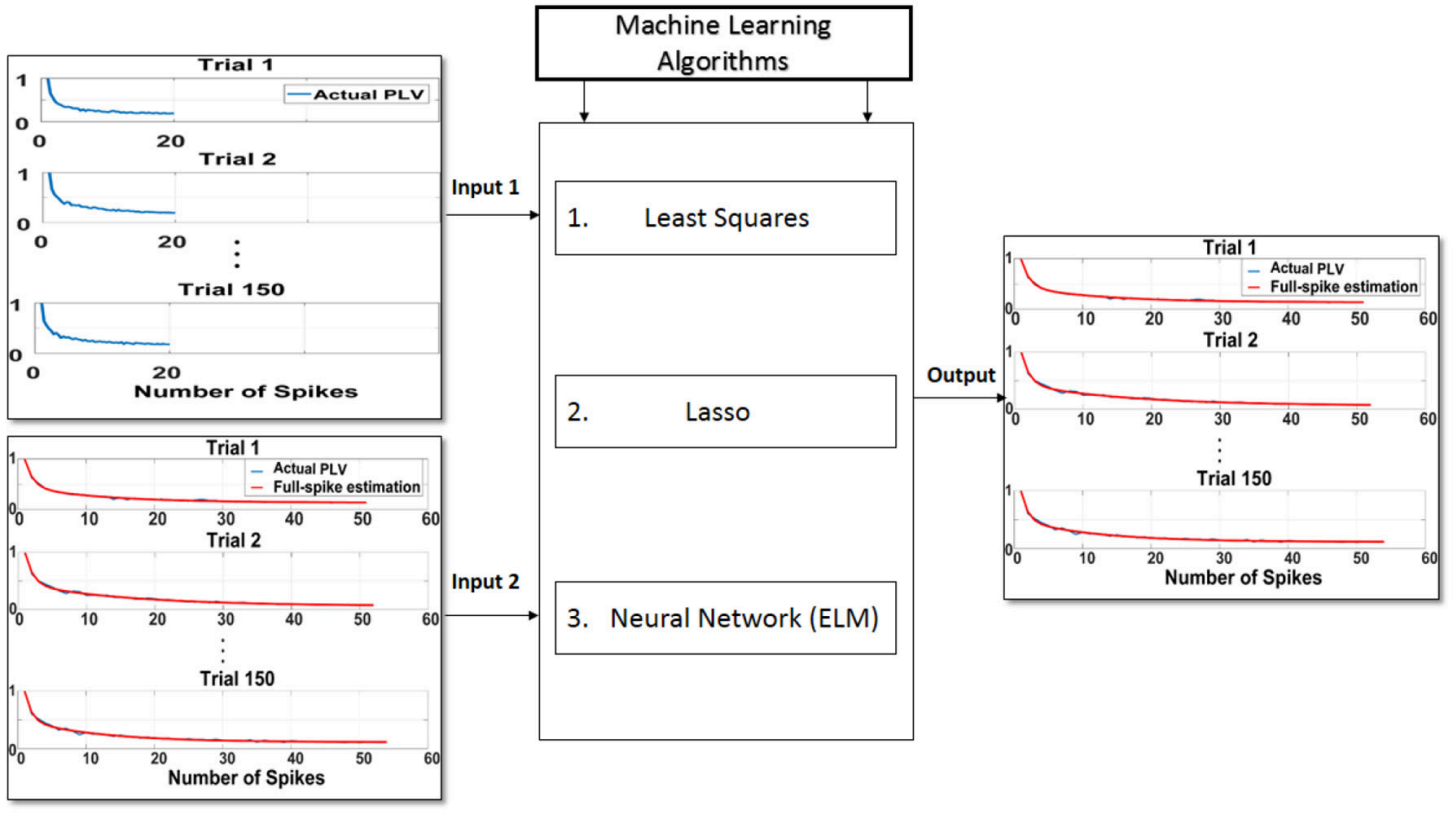
Proposed scheme to predict ideal spike-LFP phase coupling (SPC) based on the trend of the first 20 points (SR = 20) of trials and the ideal SPC of each trial. In the training phase, the first 20 points of each 150 trials as input 1, along with the asymptote of each of the trials or ideal SPC as input 2, are designated as the inputs to the machine learning algorithms of least squares, Lasso, and ELM. The output of the learning system or the corrected SPC is shown to be quite close to the ideal SPC. This model is trained based on the trend of the first 20 points of trials and the ideal SPC of each trial.

In the second step, training phase for the machine learning algorithms is illustrated using the least squares, Lasso, and ELM algorithms and depicted in Figure 3. To this end, the hold-out validation method was applied and the procedure was repeated 100 times. In fact, 200 trials were selected according to the 100 neurons as primary trials, and for each iteration, 150 trials were selected randomly for the training phase and the rest of trials were chosen for the test phase. In order to obtain higher accuracy for the training phase, trials with higher spikes are selected. We attempted to obtain an average spike rates for all neurons but noticed that there were not too many trials with at least 70 spikes. Therefore, 200 trials were chosen among 100 neurons which had at least 70 spikes. In the training phase, the first 20 points (SR = 20) of each 150 trials along with the asymptote of each of the trials (ideal SPC) are depicted as the inputs of the machine learning algorithms, namely inputs 1, 2 of Figure 3. The output of the learning system or the corrected SPC is shown to closely follow the ideal SPC with minor error and high accuracy. This model is trained based on the trend of the first 20 points of trials and the ideal SPC of each trial. Therefore, to determine the minimal number of spikes or points, it is necessary to obtain the average number of spikes for each trial. Using this average, it is possible to estimate the number of SRs. The advantage of this model is that a set number of spikes, in this case SR = 20, can easily be selected in almost every trial as the minimum number of spike occurrence.

**Figure 4:**
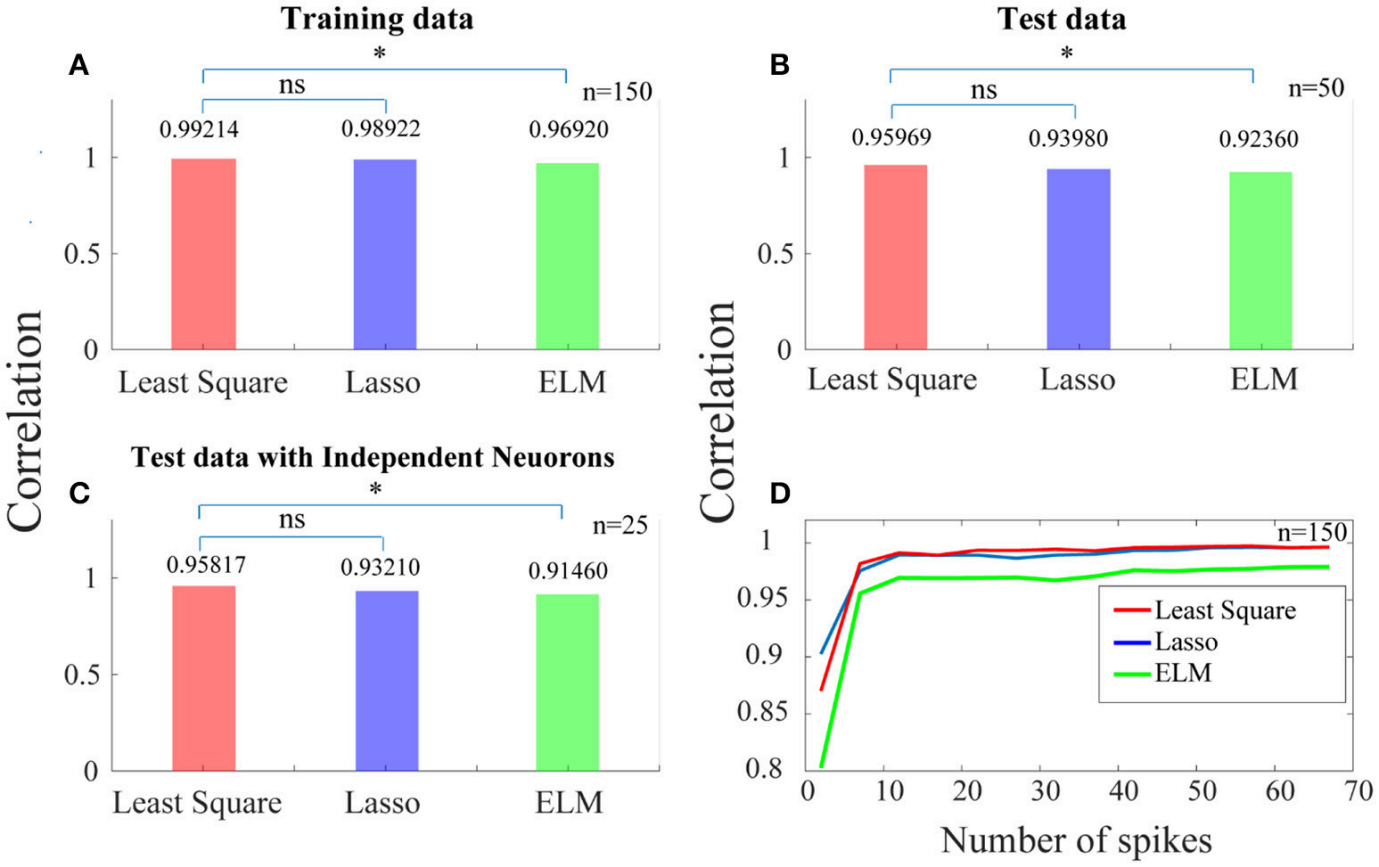
Predicting a more reliable SPC for trials with small spike rates. **(A)** Each of the bars shows the correlation between the ideal SPC and the corrected SPC across 150 trials. The correlation is performed using three separate algorithms of least squares, Lasso, and ELM (the red, blue, and green bars, respectively). **(B)** Shows that the corrected SPC can accurately predict the ideal SPC based on the first 20 points of each test trials with a high correlation. **(C)** Shows the correlation between the ideal SPC and the corrected SPC for 25 trials which are selected from 25 independent neurons. Least squares approach has the best performance, with a correlation of 0.99214, 0.95969, and 0.95817, for **(A–C)**, respectively. **(D)** Shows the correlation between the ideal and the corrected SPC curves which is modeled based on different spike rates across 150 trials. Statistically, the least squares algorithm is significantly different from the ELM algorithm (*p* < 0.05^*^; sign test) while it is not significantly (“ns”) different from the Lasso algorithm.

In Figure 4A, each of the bars shows the correlation between the ideal SPC and the corrected SPC, which is either based on trained SPC or on output of the training phase. As this correlation is very high, it indicates that the training phase is appropriately implemented. The correlation is performed using least squares, Lasso, and ELM independent algorithms illustrated as red, blue, and green bars in Figure 4, respectively. It is evident that a very high correlation between the ideal SPC and the corrected SPC is achieved. Furthermore, comparing the performance of the three algorithms shows that the least squares has the best performance, with a correlation of 0.99214.

In the test phase, 50 trials (50 test trials are independent of 150 training trails) are used and the first 20 points of each of these trials are only used as the input to the trained model. Figure 4B shows that the corrected SPC can accurately predict the ideal SPC based on the first 20 points of each test trials with a high correlation. The results of this section are also obtained using the three aforementioned algorithms, and are similar to the training phase, where the best result is obtained by least squares, with a correlation of 0.95969.

In previous steps, we used 150 trials as the training data and 50 trials as the test data. Therefore, it is possible to have more than one trial from a neuron in our analyses. In order to have only one trial from a neuron, we have to set a constraint for selecting the trials in the test phase. That is, the test phase trials must be from independent neurons. The purpose of this stage is to check whether there exit a high correlation between the ideal and the corrected SPC, when the trials are derived from independent neurons and are used as the test data. Figure 4C shows the correlation between the ideal SPC and the corrected SPC for 25 trials which are selected from 25 independent neurons. The results show that the corrected SPC can well follow the ideal SPC with high correlation in all three algorithms. Also, the best answer is obtained by least squares, with a correlation of 0.95817.

Figure 4D shows the correlation between the ideal and the corrected SPC curves which is modeled based on the different number of points (between the first points from 1 to 100) of 150 trials. Different points (SR = 20) are evaluated for each of the 150 trials using all three algorithms. As it is shown in Figure 4D, for trials with a spike rates higher than 10, the correlation is very high between the ideal and the corrected SPC. Also, as the number of spikes increases, the correlation increases as well. We also used a statistical test (sign test) for quantitative comparisons between least squares, Lasso, and ELM algorithms. According to the statistical test outcome, it was observed that the least squares algorithm was significantly different from the ELM algorithm (*p* < 0.05^*^; sign test) while it was not significantly (“ns” as depicted in Figure 4) different from the Lasso algorithm.

Furthermore, we applied the R-squared criterion (*R*^2^) as a statistical measure that indicates the model's goodness of fit. Table 1 depicts the Goodness of fit (*R*^2^) in order to confirm the proposed scheme's ability to predict the ideal SPC.

**Table 1:** Goodness of fit [R-squared criterion (*R*^2^)] is presented to confirm the proposed scheme's ability to predict ideal spike-LFP phase coupling (SPC).

**Validity criterion**	***R*****-squared**		
**Training and test phases**	**A**	**B**	**C**
Least squares	0.9563	0.8842	0.8532
Lasso	0.9314	0.8262	0.8129
ELM	0.9162	0.8123	0.7910

*Column A represents R^2^ for the training phase, column B depicts R^2^ for the test phase, and column C shows R^2^ for the test phase with independent neurons. The R-squared is performed using three separate algorithms of least squares, Lasso, and ELM*.

**Figure 5:**
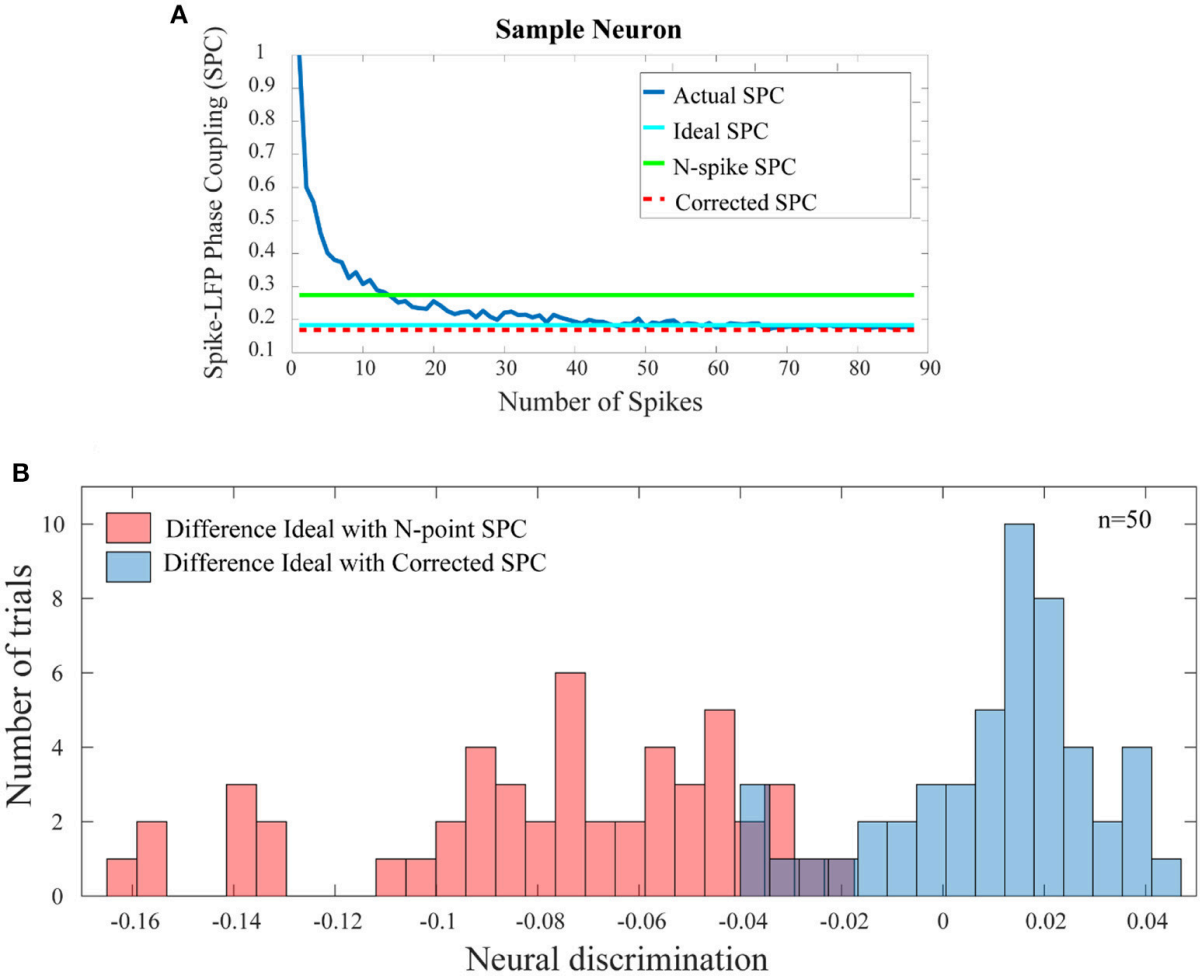
Comparison of the error between the SPC based on “N-spike” and proposed corrected SPC with the ideal SPC. **(A)** For a sample trial of neuron, shows that the corrected SPC is very close to the ideal SPC. The difference between the SPC based on the N-spike (green line) and the ideal SPC (turquoise line) is much higher than the difference between the corrected SPC (dash red line) and the ideal SPC. **(B)** Depicts the histogram distribution of the difference between the N-spike SPC and the ideal SPC, as well as the difference between corrected SPC and ideal SPC, across 50 test trials (*p* < < 0.001; sign test).

Figure 5A shows the comparison results for a sample neuron. The results presented in Figure 5A are obtained based on trained model in which only 20 primary spikes or trend of the first 20 points are used to predict ultimate or ideal value of SPC. This calculated number is shown as a horizontal line which intercepts the SPC axis at the final value of corrected SPC. As depicted, the corrected SPC is quite close to the ideal SPC and while there is very small error, the measure of the SPC is proved to be accurate. As mentioned before, the PLV is biased on the spike rates and equalizes the spikes at a specified rate. Thus, to calculate the SPC by the PLV method, an equalizing scheme is used which is based on a threshold T of number of spikes. Therefore, the trials with number of spikes below T are deleted, and those with higher number of spikes, are equalized to T. Here, we define a spike N (“N-spike”) as the threshold for which the spike rates are equalized and therefore associate this value with the PLV based SPC measure. Figure 5A compares the proposed method with the PLV based approach, which clearly shows the superiority of the proposed approach.

Figure 5B shows the histogram distribution of the difference between the N-spike and ideal SPC as well as the SPC and ideal SPC, across 50 test trials (*p* < < 0.001; sign test). Therefore, the model-based estimation for the SPC provides a plausible improvement as compared to the simple calculation of the SPC by the PLV method.

### A bias-free framework for SPC

**Figure 6:**
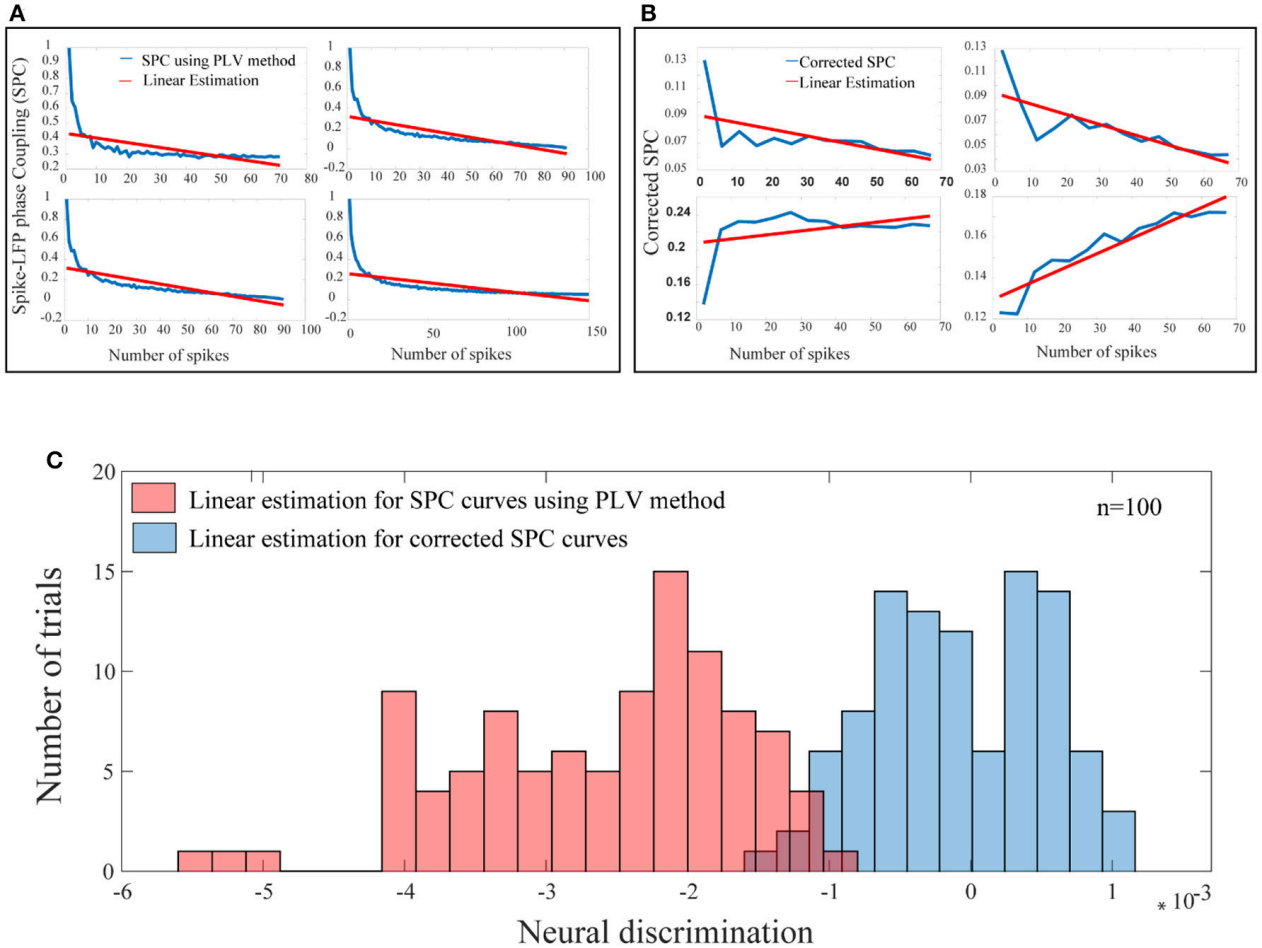
A bias-free framework for SPC and comparing the proposed framework with the conventional method of PLV. **(A)** For a sample of 4 neurons, these curves are provided based on the PLV method with no correction. There is a systematic relation of strictly descending between the spike rates and the PLV (blue curves). The red lines show the linear estimations. **(B)** For a sample of 4 neurons, for each of the test trials and for each of their spike rates, the value of the corrected SPC (blue curves) is computed. The red lines show the linear estimations. **(C)** The blue histogram shows the distribution for all linear estimations across 100 trials among 100 neurons which suggests no systematic relation between the spike rates and the corrected SPC and is symmetrically distributed about zero. The red histogram shows the distribution for all linear estimations across 100 trials among 100 neurons which suggests a systematic relation between the spike rates and the PLV, and is strictly descending (*p* < < 0.001; sign test).

In the third step, for each test trial and for each of their spike rates, the values of the corrected SPC are computed. It should be noted that, the trained model and the test trials must be based on the same spike rates. These results are presented in Figure 6. Also, in Figure 6 our proposed method and the PLV or conventional method are compared. In Figure 6A, the blue curves depict PLV based method for a sample of 4 neurons and illustrate a systematic and strictly descending spike rates and SPC. This result is an expected behavior of the PLV-based method.

The blue curves in Figure 6B represent the results of the third phase for a sample of 4 neurons. As an example for a given test trial, in order to calculate the corrected SPC for the first 20 points (SR = 20), we should use the trained model associated with the same spike rate. This process is performed for each rate of spikes in each of the 100 trials. Figure 6B depicts proposed corrected SPCs for different spike rates. That is, each point on the curve is a corrected SPC for the correspondent value on spike rate axis. For example, if there are spike rates from 1 to 70, the corrected SPC process is repeated 70 times. In fact, for each spike rate, a separate machine learning algorithms is trained correspondent to that spike rate. Through this approach, a unique corrected SPC is obtained that forms one point in the “corrected SPC-spike rate” chart. Applying corrected SPCs for corresponding spike rates leads to the blue curve in Figure 6B. As illustrated, it is obvious that, one can expect descending, ascending, or flat curves while SPCs are corrected. In other words, our proposed method corrects each SPC separately to achieve a corresponding point in the corrected SPC curve. In both Figures 6A,B, the red lines depict the linear estimations which are applied to each of the above mentioned methods.

Figure 6C illustrates the distribution functions for linear estimations based on the proposed method (blue histogram) which suggests no bias and is symmetrically distributed around. The red histogram in the Figure 6C illustrates linear estimations based on the PLV method which illustrate a systematic bias with strictly descending behavior (*p* < < 0.001; sign test).

## Discussion

Measuring the coupling of single neuron's spiking activities to the LFP is a method which focuses on neuronal synchronization and locking the spiking activity to the LFP is a feature of this inter-neuronal synchrony. Several SPC synchronization measures have been introduced in previous studies (Tibshirani, 1996, 1997; Fries et al., 2002; Abdi, 2007; Curtis et al., 2009; Grasse and Moxon, 2010; Hagan et al., 2012). This study proposed a new framework to predict a more reliable SPC through modeling and using machine learning algorithms for neurons with low spike rates. Results show that the proposed framework is considerably more accurate than the previous methods. Also, in this study we introduced a bias-free framework for the SPC and compared it with the conventional methods such as the PLV. Furthermore, it is shown that the proposed framework has the general capability to correct for the bias on spike rates.

As noted before, currently the main synchronization measures are the PLV (Lachaux et al., 1999), SFC (Grasse and Moxon, 2010), PPC (Vinck et al., 2010), and SCMS (Li et al., 2016). The main characteristics of these synchronization measures are as follows.

PLV computes the magnitude of mean phase difference between the LFP and the spikes as the strength of the SPC. The shortcoming of this method is its dependence on the spike rates. Studies that use this method, equalize the spikes at a specific rate. Thus, to calculate the SPC by the PLV method an equalizing scheme is used for the spikes based on a threshold T.

SFC measures the synchronous activity between the LFPs and the spike rates as a function of frequency. It is computed by comparing the magnitude of the frequency in the STA and the average magnitude of the frequency for each LFP segments that is involved in the STA. Furthermore, a spike which occurs during a high magnitude cycle of the LFP will have more impact on the value of the SFC than a spike which occurs during a low magnitude LFP cycle.

PPC computes the mean inter-spike similarity of the LFP phase across all the possible pairs of spikes. However, the PPC is unbiased only for very high spike rates while measuring the SPC for short trials is considered to be a challenge for many studies. Furthermore, PPC results in negative values for some cases, which cannot be justified physiologically.

In the SCMS, each spike is placed in the center of the time window with duration T. At the first step, LFP segments are obtained by the PLV method and accordingly, the SCMS calculates the point-to-point phase difference between each pair of these LFP segments. Similarly, this process is done for all other LFP segment pairs and as a result, a correlation matrix is formed. The eigenvalues of this matrix must be calculated and the maximum eigenvalue is reported as a measure of synchronization. Accordingly, SCMS measures the correlation in terms of phase in regions around the spikes, dictated by the length of the LFP segments, and not at the moment of the spike occurrence. Such behavior distinguishes the SCMS method from the SPC.

This study proposed an approach which overcomes the aforementioned problems through the following steps.

### Estimation of the ideal SPC value using mathematical modeling

The ideal SPC was estimated by a modeling scheme which is based on two exponential functions and the asymptote of the fitted model to each of the SPC curves. The average and error bar of RMSE across 100 trials among 100 neurons were shown to be quite small (0.0003±0.01). This suggests that the exponential model accurately represents the SPC curve.

### Predicting a more reliable SPC by machine learning algorithms for trials with small spike rates

Training and test phases were performed using a proposed approach based on three separate algorithms, namely least squares, Lasso, and ELM. This model was trained based on the trend of the first 20 points (SR = 20) of each trial. The advantage of this method is that the number of spikes is made possible in trials with the lowest spike rates. In the test phase, the model accurately predicted the ideal SPC based on the first 20 points of each test trials. Comparing the results of the three algorithms showed that the least squares had the best performance, with a correlation of %96.

### A bias-free framework for SPC as compared with conventional methods such as PLV

For each test trial and for each of their spike rates, the value of the proposed corrected SPC was computed. Hence, a linear estimation was applied to each of the corrected SPC curves. The distribution function for all linear estimations across test trials was obtained, which suggested no bias in the corrected SPC. Furthermore, the *p*-value was calculated for these results using the statistical sign test that demonstrated its statistical significance (*p* < < 0.001; sign test).

In summary, this study proposed a new framework for predicting a more reliable SPC for neurons with low spike rates. As a result, due to absence of bias on the spike rates, there is no need for equalizing the number of spiking neurons, and therefore the related information is preserved. Furthermore, this framework has the general capability to correct for the bias in the spike rates for various methods.

## Author contributions

MZ contributed to the concept of the work, analysis of data, and draft of the manuscript. MJ contributed to the concept of the work, supervising the analysis, and revising and finalizing the manuscript. MD contributed to the concept of the work, supervision, and revising the manuscript.

### Conflict of interest statement

The authors declare that the research was conducted in the absence of any commercial or financial relationships that could be construed as a potential conflict of interest. The handling Editor declared a shared affiliation, though no other collaboration, with one of the authors MD.
